# Screening and Detecting *Salmonella* in Different Food Matrices in Southern Tunisia Using a Combined Enrichment/Real-Time PCR Method: Correlation with Conventional Culture Method

**DOI:** 10.3389/fmicb.2017.02416

**Published:** 2017-12-07

**Authors:** Mariam Siala, Amina Barbana, Salma Smaoui, Salma Hachicha, Chema Marouane, Sana Kammoun, Radhouane Gdoura, Férièle Messadi-Akrout

**Affiliations:** ^1^Department of Biology, Preparatory Institute for Engineering Studies of Sfax, University of Sfax, Sfax, Tunisia; ^2^Department of Life Sciences, Research Laboratory of Environmental Toxicology-Microbiology and Health (LR17ES06), Faculty of Sciences of Sfax, University of Sfax, Sfax, Tunisia; ^3^Regional Hygiene Care Laboratory, Department of Microbiology, Hedi-Chaker University Hospital, Sfax, Tunisia; ^4^Department of Biology B, Faculty of Pharmacy of Monastir, University of Monastir, Monastir, Tunisia

**Keywords:** *Salmonella*, *invA* qPCR, prevalence, detection, different food matrices, serotype

## Abstract

A combined enrichment/ newly developed *invA* TaqMan^®^ real-time PCR (qPCR) method as a screening assay to detect *Salmonella* spp. in 500 naturally food matrices is evaluated. DNA template for qPCR was extracted from an overnight pre-enriched sample in buffered peptone water using lysis–guanidine isothiocyanate method. Heterologous internal amplification control (IAC) was incorporated during qPCR assays and co-amplified with the *invA* gene of the target pathogen. *InvA* qPCR exhibited 100% specificity when testing 94 *Salmonella* strains (inclusivity) and 32 non-*Salmonella* strains (exclusivity). The qPCR showed a consistent detection of two copies of the *invA* gene/PCR reaction, a good intra- and inter-run reproducibility with a good PCR efficiency (89.6%). QPCR was sensitive and showed *Salmonella* detection at 8.5 × 10^0^ CFU mL^-1^ of artificially spiked poultry meat -BWP solution in less than 40 cycles. When analyzing 500 different food matrices and comparing the results with the ISO 6579:2002 conventional culture method, the sensitivity and specificity were 100 and 76.6%, respectively. QPCR showed *Salmonella* spp. DNA in raw poultry meat 27/45 (60%), milk 31/93 (33.3%), raw red meat 5/13 (38.5%), and fish 11/46 (23.9%) samples. The prevalence of *Salmonella* spp. in cakes, dairy, cooked meals, charcuterie products using qPCR was 11/14 (26.8%), 5/22 (22.7%), 32/150 (21.3%), and 5/20 (25%), respectively, compared to 0% as demonstrated by culture. *S.* Anatum was the most common serovar found associated with red meat compared to *S.* kentucky isolated from fish and poultry meat. In conclusion, our study is the first to use a combined enrichment/*invA* qPCR method as a screening assay to detect *Salmonella* DNA in different types of commercialized food in Southern Tunisia. QPCR results indicate that *Salmonella* contamination is common in milk and in other types of food samples.

## Introduction

Salmonellosis is a common cause of mortality and morbidity due to water and food borne infections in almost all countries causing human gastroenteritis and typhoid fever (Malorny et al., 2008). Food sources of *Salmonella* included mainly milk, eggs, meat (poultry, beef) vegetables, and fresh fruits (Almeida et al., 2013).

To limit food borne illness worldwide due to *Salmonella* spp., improving the monitoring and control methods is necessary (Almeida et al., 2013). Conventional laboratory methods frequently used to detect *Salmonella* in foods are laborious, time consuming and allowed the detection of only higher levels of *Salmonella* (10^2^–10^3^ CFUg^-1^ or 10^2^–10^3^ CFUmL^-1^). Nevertheless, low numbers of *Salmonella* in food could present a public health problem due to the low infective dose that could be lower than 15–100 CFU (Almeida et al., 2013). Thus, fluorescent-probe-based TaqMan^®^ real-time PCR (qPCR) systems have been developed in the last decade to detect *Salmonella* nucleic acids in food samples. These latter proved a high speed, high sensitivity and specificity, as well as dispensable post-PCR steps thus reducing the risk of cross-contamination (Schuman et al., 2007; Almeida et al., 2013).

It is important, that techniques used to monitor *Salmonella* in foods have the capacity to amplify viable low levels of target bacterial specie, as well as those that are prone to stressful conditions within the food and/or during food processing (Fratamico, 2003*)*. However, there are challenges associated with qPCR using TaqMan^®^ assay, mainly a good sensitivity (detecting low levels of target pathogens), avoiding the amplification of dead cells and the inhibition effect of food components. Overcoming those challenges could be achieved by using the qPCR from the pre-culture enrichment broths of the contaminated food (Fratamico, 2003; Bohaychuk et al., 2007).

Recent studies developed molecular qPCR methods for *Salmonella* detection in food using the *invA* gene as candidate target (Hein et al., 2006; Malorny et al., 2007; Anderson et al., 2011; Li et al., 2012; Zheng et al., 2014). However, the analytical performance of *invA* qPCR for *Salmonella* detection in food is questionable since they differed in the sensitivity of used primers, the lack of use of the internal amplification control (IAC) DNA sequence to confirm the integrity of the reagents and monitor the absence of inhibitors in the sample and a limited strain panel tested for specificity of the method (Fratamico, 2003; Wolffs et al., 2006; Bohaychuk et al., 2007). Additionally, most validated studies have used a single food spiked with *Salmonella* and few validation methods have focused on a method that was used to survey the presence of *Salmonella* associated with different food matrices. Of note, there is no molecular study using an optimized qPCR as a screening tool for the detection of *Salmonella* in pre-culture enrichment broths of the naturally contaminated food in developing countries such as Tunisia. Thus, our aims were (i) to detect *Salmonella* spp. from direct food product samples by combining a pre-enrichment stage with a newly developed *invA* TaqMan^®^ qPCR assay (ii) to compare *invA* qPCR with a standard culture procedure for *Salmonella* detection in 500 commercialized food samples in South Tunisia.

## Materials and Methods

### Samples Collection and Conventional Culture for *Salmonella* spp. Isolation by ISO 6579

A total of 500 food samples were collected randomly from supermarkets within 4 months during the year 2014. Raw milk was obtained from a dairy farm located in Sfax state, Tunisia. The different food samples were transported under complete aseptic conditions in an icebox within 2 h to the regional hygiene care laboratory of Sfax (Southeast of Tunisia) for processing. These samples represented the following products: Cooked dishes (*n* = 150), milk (*n* = 93), dairy products (*n* = 22), fresh fruit, and vegetables (*n* = 70), seafood (*n* = 46), raw poultry meat (*n* = 45), cakes (*n* = 41), charcuterie products (*n* = 20), and raw red meat (*n* = 13). Fresh fruit and vegetables included raw mixed vegetables salad, fruits, and vegetables such as onion and parsley, basil and coriander. The seafood included fishes, clams, and shrimps. The poultry set included samples of chicken and turkey meat, poultry legs, and wings. The red meat was composed of beef meat and liver samples. The charcuterie products were composed of salami and sausage samples.

Foods were analyzed within 24 h of sample receipt. The detection of *Salmonella* was processed using conventional culture-based methods according to the International Organization for Standardization [ISO] (2002) protocol (ISO 6579:2002). Briefly, a total of 25 g or 25 mL of sample was homogenized with 225 mL of Buffered Peptone Water (BPW) (Oxoid, Basingstoke, United Kingdom), stomached for 45 s and followed by incubation at 37°C for 22 h. After pre-enrichment, 100 μl and 1 mL samples were taken and mixed with 10 ml of Rappaport Vassialidis soya (RVS) broth (BD Difco, Germany) and Muller Kauffmann tetrathionate-novobiocin (MKTTn) broth (biokar, France), respectively. Cultures were incubated overnight at 37°C fo MKTTn broth and at 42°C for RSV broth. After the selective enrichment step, a loopful of each enriched sample was streaked on differential medium [Xylose Lysine Desoxyscholate (XLD) and Hoektoen]. Suspected colonies were identified biochemically (urea, kligler hajna, ONPG) and then confirmed with Api 10S (bioMérieux, United States) and serologically as described below.

One mL of pre-enrichment culture in BPW was subjected to preparation of DNA template for *Salmonella* spp. qPCR assays.

### Serological Identification

Serotyping was performed at the National Center for Enteropathogenic Bacteria, Pasteur institute, Tunis. Briefly, serology was done using slide agglutination tests with commercial predefined polyvalent and monovalent somatic and flagellar antisera according to Kauffmann–White serotyping scheme (Grimont and Weill, 2007).

### Preparation of Template DNA for qPCR

An aliquot of 1 mL of pre-enriched cultures was centrifuged at 15,000 × *g*, 5 min. The collected pellet was used for DNA extraction using the lysis–guanidine isothiocyanate (GuSCN) method as described previously (Kawasaki et al., 2010). Two microliters of the solution was used as template for the qPCR.

### Real-Time PCR for Detection of *Salmonella* spp.

#### *Inv A* Primers and Taqman Probes Design

Primers (F:5′-ACAGTGCTCGTTTACGACCTGAAT-3′,R:5′-AGACGGCTGGTACTGATT ATAAT-3′) and TaqMan probe (5′-Fam-CGA-CCC-CAT-AAACACCAATATCGCC-BHQ1b-3′) sequences were designed using Primer3 software^1^ (Rozen and Skaletsky, 2000) to amplify 243 bp of *Salmonella* spp. *invA* gene. They were examined to exhibit optimal biophysical properties, no dimer formation with Oligoanalyzer 3.1. They were blasted against the nucleotide database of the NCBI website^2^ to ensure identity among reported BLAST sequences for the target gene and the absence of significant homology with other microorganism sequences. The probe and primers were purchased from Integrated DNA Technologies (IDT, Coralville, IA, United States). The probe was labeled with a fluorescent reporter dye, 6-carboxyfluorescein (FAM), on the 5′-end and with Black Hole Quencher^®^ (BHQ) at the 3′ end of the probe.

#### Exogenous Internal Amplification Control (IAC)

An exogenous DNA internal amplification control (my-IAC) was incorporated in all samples during qPCR assays. The (my-IAC) heterologous DNA sequence, primers, and probe used in this work were described previously (Deer et al., 2010) and were kindly provided by Narjol Gonzalez Escalona (FDA, CFSAN). The (my-IAC) DNA sequence is a synthetic construct that does not match any currently available sequence in the GenBank database^2^ (Deer et al., 2010; Kasturi and Drgon, 2017). My-IAC probe was labeled at the 5′- end with Cy5 dyes as reporter and at the 3′- end with Iowa Black RQ-Sp as quencher. My-IAC DNA was serially diluted to establish the optimal concentration giving a positive signal at a *Ct* > 25 that could be accurately detected in the presence of *Salmonella* DNA.

#### *Salmonella* spp. Real-Time PCR

The qPCR was run in a taqman mastermix containing 10 μl of Ex Taq Premix Tli RNaseH Plus (Takara, Japan), 250 nM of each *invA* primer, 100 nM of *invA* probe, and 2 μl of purified DNA in a final volume of 20 μl using nuclease-free water. Regarding the IAC, 320 nM IAC primers, 160 nM IAC probe, and 0.5 μl of IAC DNA template (0.5 pg μl^-1^, approximately 10^2^ to 10^3^ copies per PCR reaction) were added per reaction as described previously (Deer et al., 2010). CFX96TM real-time PCR thermocycler (Biorad, France) system was used for all the qPCR experiments. The optimal qPCR efficacy was achieved using cycling profile including a denaturation at 95°C for 30 s, followed by 40 cycles of 05 s at 95°C and 30 s at 60.0°C. Each qPCR included one positive control (*S. enterica* DNA), no template control (mastermix and sterile water) and a negative DNA control (*Escherichia coli* DNA). All qPCR experiments were performed in duplicate.

#### Analytical Specificity of qPCR

DNA extracted from *Salmonella* isolates (*n* = 94) (**Table 1**) and strains of other genera (*n* = 32) including those of the family *Enterobacteriaceae*, such as *E. coli, Shigella*, and *Klebsiella pneumoniae* (**Table 2**) were used to test the specificity of the designed *invA* primers and probe used in this study.

**Table 1:** *Salmonella* strains used for specificity testing.

Species (*N*°)	Number of strains tested	Origin/accession number	PCR amplification	
			*invA*	IAC
*Salmonella. enterica*	1	–/*ATCC 43972*	Positive	Positive
*Salmonella* Typhi	1	Human/*ATCC 19430*	Positive	Positive
*Salmonella* spp.	1	Clinical isolate^∗^/–	Positive	Positive
*Salmonella* spp.	18	Chicken/–	Positive	Positive
*Salmonella* Enteritidis	1	Clinical isolate/–	Positive	Positive
*Salmonella* Infantis	1	Clinical isolate/–	Positive	Positive
*Salmonella* Kentucky	15	Chicken, milk/–	Positive	Positive
*Salmonella* Brandenburg	1	Beef meat/–	Positive	Positive
*Salmonella* Muenchen	1	Clam/–	Positive	Positive
*Salmonella* Schwarzengrund	3	Chicken, Fish/–	Positive	Positive
*Salmonella* Typhimurium	4	Bovin, Clam^∗^/–	Positive	Positive
*Salmonella* Newport	3	Chicken, milk/–	Positive	Positive
*Salmonella* London	2	Clam/–	Positive	Positive
*Salmonella* Anatum	5	Clam, milk/–	Positive	Positive
*Salmonella* Irenea	2	Clam/–	Positive	Positive
*Salmonella* Enteritidis	14	Chicken, clam/–	Positive	Positive
*Salmonella* Enteritidis	1	–/*NCTC 13349*	Positive	Positive
*Salmonella* Salamae	1	–/*ATCC 43972*	Positive	Positive
*Salmonella* Poona	1	Clam/–	Positive	Positive
*Salmonella* Matadi	1	Clam/–	Positive	Positive
*Salmonella* Brandcaster	1	Clam/–	Positive	Positive
*Salmonella* Bredeney	1	Clam/–	Positive	Positive
*Salmonella* Montevideo	1	Clam/–	Positive	Positive
*Salmonella* Montevideo	8	Red meat/–	Positive	Positive
*Salmonella* Frintrop	1	Clam/–	Positive	Positive
*Salmonella* Onderstepoort	1	Clam/–	Positive	Positive
*Salmonella* Saphra	1	Clam/–	Positive	Positive
*Salmonella* Zanzibar	1	ND/–	Positive	Positive
*Salmonella* Schwarzengrund	1	Clam/–	Positive	Positive
*Salmonella* Zanzibar	1	ND/–	Positive	Positive
	Total = 94			

*ATCC: American Type Culture Collection, Manassas, VA, United States. ND: not determined*.

**Table 2:** Non *Salmonella* strains used for specificity testing (all tested strains are negative for the *invA* gene).

Species (*N*°)	Number of strains tested	PCR amplification	
		*invA* gene	IAC
*Escherichia coli (*ATCC 8739)	1	Negative	Positive
*E. coli (*Lab isolate)	4	Negative	Positive
*Listeria monocytogenes* ATCC 19115	1	Negative	Positive
*Listeria monocytogenes* (Lab isolate)	1	Negative	Positive
*Listeria innocua* (Lab isolate)	1	Negative	Positive
*Citrobacter* spp. *(*Lab isolate)	1	Negative	Positive
*Klebsiella pneumoniae* (Lab isolate)	1	Negative	Positive
*Proteus mirabilis* (Lab isolate)	4	Negative	Positive
*Pseudomonas aeruginosa* ATCC 27853	1	Negative	Positive
*Enterococcus feacalis* (Lab isolate)	1	Negative	Positive
*Legionella pneumophila* (Lab isolate)	1	Negative	Positive
*Staphylococcus aureus* ATCC 25923	2	Negative	Positive
*Brucella abortus* (Lab isolate)	1	Negative	Positive
*Coxiella burnetii* (Lab isolate)	1	Negative	Positive
*Bacillus cereus* (Lab isolate)	1	Negative	Positive
*Bacillus cereus* (Lab isolate)	1	Negative	Positive
*Bacillus subtilus* (Lab isolate)	2	Negative	Positive
*Vibrio alginolyticus* (Lab isolate)	1	Negative	Positive
*Vibrio parahaemolyticus* (Lab isolate)	1	Negative	Positive
*S. flexneri* (Lab isolate)	1	Negative	Positive
*S. sonnei* (Lab isolate)	1	Negative	Positive
*Shigella* spp. (Lab isolate)	1	Negative	Positive
*C. jejeuni* (Lab isolate)	1	Negative	Positive
*C. coli* (Lab isolate)	1	Negative	Positive
	Total = 32		

*ATCC: American Type Culture Collection, Manassas, VA, United States*.

#### Detection Limit and Reproducibility of qPCR Assay

The threshold limit was determined using an amplification product of 243 bp cloned into a pGEM- T^®^ plasmid Vector system I (Promega, France) was used. The analytical sensitivity of qPCR using a 10-fold serially diluted plasmid in DNase- and RNase-free water were analyzed in a range from 2.00 × 10^0^ to 2.00 × 10^5^ gene copies per PCR reaction. In order to assess the intra- and inter-assay values of qPCR variation, DNA from a 10-fold serially diluted plasmid was subjected to qPCR in duplicate, with two different mixes performed on three different days. Intra and inter-run reproducibility was assessed by comparing mean threshold cycle (Ct) and standard error of the mean of replicates obtained in the three runs.

#### Artificially Spiked Raw Poultry Meat Sample

Raw poultry meat confirmed to be free from *Salmonella* by conventional culture and conventional PCR methods was used for the seeding experiment. Serial dilutions were made from overnight cultures of *Salmonella* Enteritidis (OD = 0.66, at 600 nm) to obtain desired cell concentration for artificially spiked sample. The precise number of CFU was confirmed using the plate count method onto nutritive agar (Oxoid, Basingstoke, United Kingdom). Then, 25 g of raw poultry meat was mixed with 225 mL of BPW and artificially inoculated with 1 mL of the appropriately diluted suspension of *Salmonella* Enteritidis. Six samples ranging from 8.5 × 10^5^ to 8.5 × 10^0^ CFU mL^-1^ of poultry meat – BPW solution were prepared. One negative (un-inoculated) sample control was also prepared and included in all experiments. After inoculation, samples were stomached for 45 s. One milliliter of sample was subjected to the DNA extraction using the method mentioned above. Each dilution was tested in duplicate.

### Statistical Analysis

The χ^2^ and Fisher’s exact 2-tailed tests were assessed and differences were considered significant at values of *P* ≤ 0.05 using SPSS 16.0 statistical software (SPSS Inc., Chicago, IL, United States).

## Results

### Analytical Specificity

Specificity of the *invA*-specific qPCR assay was accomplished by analyzing DNA samples obtained from 94 *Salmonella* (inclusivity; **Table 1**) and 32 non *Salmonella* strains (exclusivity; **Table 2**). Amplification of a 243 bp PCR product was considered a positive result. A 243 bp amplicon was obtained for all of the *Salmonella* strains and no amplicon result was observed for the non-*Salmonella* strains.

### Internal Amplification Control

The IAC DNA was added into the PCR reaction mix during all qPCR assays and was detected as expected. The results were considered valid if, a positive signal in the Cy5 detection channel on CFX96TM qPCR thermocycler was obtained.

### Detection Limit and Reproducibility of the qPCR

**FIGURE 1:**
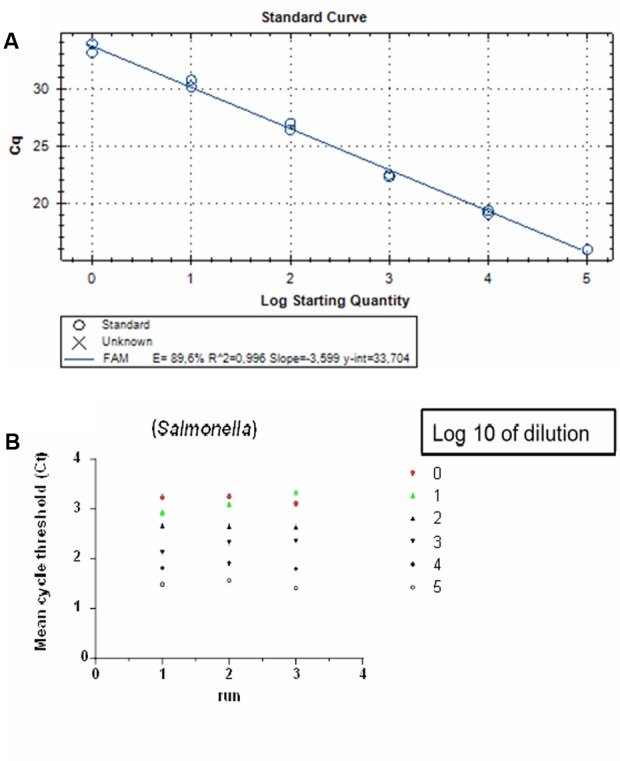
Detection limit and reproducibility of qPCR on *Salmonella* Enteritidis targeting the *invA* gene: **(A)** Standard curve generated from quantification cycle numbers of a 10-fold dilution series of an amplicon product of 243 bp cloned into into a pGEM-T^®^ plasmid vector in DNase- and RNase-free water (2.00 × 10^0^ to 2.00 × 10^5^ copies per reaction). QPCR was performed with the CFX96 TM real-time PCR system. **(B)** Inter and intra-run reproducibility assessed using 2.00 × 10^0^ to 2.00 × 10^5^ copies per reaction in three independent runs.

Before applying our qPCR assay to food samples, a prior determination of the linear range and detection level was assessed using a known numbers of cloned PCR targets. A pre-enrichment step was not included in this experiment. The detection level was determined to be two copies of the *invA* gene/PCR, which was the lowest number tested, and the standard curve was linear over the tested quantity ranged from 2.00 × 10^0^ to 2.00 × 10^5^ gene copies/PCR (**Figure 1A**). The amplification efficiency of the qPCR reaction was calculated to be 89.6% and was obtained from the slope of this standard curve (range: 2.00 × 10^5^ to 2.00 × 10^0^ copies of the *invA* gene equivalents per PCR reaction). Intra- and inter-run reproducibility was high for *Salmonella* spp. *inv*A qPCR (**Figure 1B**). The average difference between tenfold dilution was 3.47 cycles when testing the *Salmonella* spp. DNA positive controls.

### *Salmonella* Detection in Spiked Raw Poultry Meat Sample

**FIGURE 2:**
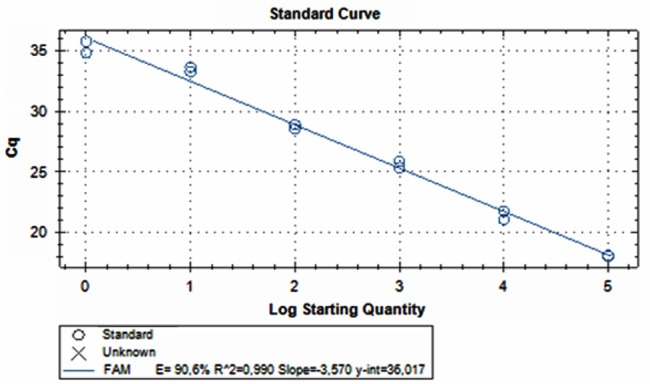
Amplification of DNA extracted from artificially 10-fold serial inoculated raw poultry meat sample, ranging from 8.5 × 10^5^ to 8.5 × 10^0^ CFU mL^-1^ of poultry meat-BWP solution using qPCR.

Amplification of DNA extracted from artificially 10-fold serial inoculated raw poultry meat sample, showed that qPCR gave a positive result from 8.5 × 10^5^ to 8.5 × 10^0^ CFU mL^-1^ of poultry meat-BWP solution using qPCR in less than 40 cycles (**Figure 2**). The *C*t values of the experiment ranged from 18 ± 0.28 to 35.27 ± 0.70.

### Isolation and Biochemical Identification of *Salmonella* spp. in Different Types of Food Samples Using ISO 6579

**FIGURE 3:**
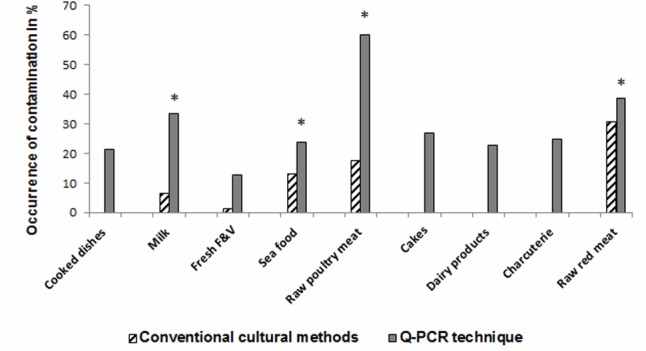
Occurrence of *Salmonella* in different types of food matrices. ^∗^Indicates significant differences between *Salmonella* positivity by culture compared to qPCR (*P* ≤ 0.05).

**FIGURE 4:**
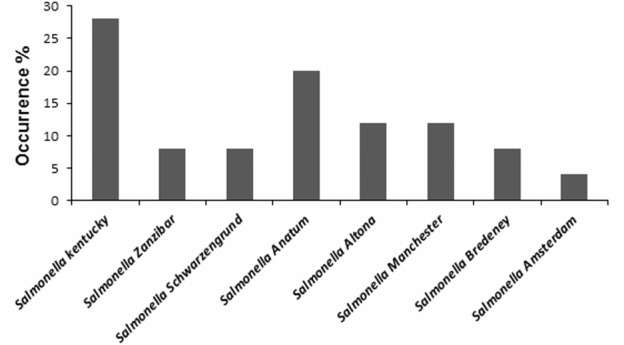
Occurrence of different *Salmonella* serotypes isolated from food matrices.

A total of 500 food samples which were collected from local markets were processed during the study period. Out of these, 25 samples (5%) were found positive for *Salmonella* by conventional isolation and identification methods recommended by ISO 6579:2002 (**Table 3**). *Salmonella* spp. were isolated from milk (6/93, 6.4%), fish (6/46, 13%), and raw vegetable salad (1/70, 1.4%). The majority of isolates originated from raw red meat (4/13, 30.7%) and raw poultry meat (8/45, 17.8%) samples (**Figure 3**). Among the 25 *Salmonella* isolates, a total of eight serotypes were identified. **Table 4** depicts the different *Salmonella* serotypes isolated from the raw poultry meat, raw red meat, fish, milk, and raw vegetable salad samples. Comparison of the prevalence of the eight serotypes is shown in **Figure 4**. *S.* kentucky, *S.* Anatum, *S.* Altona, and *S*. Manchester were the most frequent serotypes, accounting for 28, 20, 12, and 12%, respectively, of all isolates. The other *Salmonella* serotypes including *S.* Zanzibar, *S.* Schwarzengrund, and *S*. Bredeney showed the same prevalence of 8%. *S*. Amsterdam was isolated less frequently (4%).

**Table 3:** The results of *Salmonella* spp. detection by conventional cultural methods and qPCR techniques.

	*Salmonella* detection positivity	
Type of food samples No,	Conventional cultural method	Q-PCR technique	^∗^χ^2^ test (*P*)
	Positivity, (%) No,	Positivity, (%) No,	
Cooked dishes (*n* = 150)	0 (0/150)	21.3 (32/150)	–
Milk (*n* = 93)	6.4 (6/93)	33.3 (31/93)	0.001
Fresh fruit and vegetables (*n* = 70)	1.4 (1/70)	12.8 (9/70)	–
Sea food (*n* = 46)	13 (6/46)	23.9 (11/46)	≤0.001
Raw poultry meat (*n* = 45)	17.8 (8/45)	60 (27/45)	0.014
Cakes (*n* = 41)	0 (0/41)	26.8 (11/41)	–
Dairy products (*n* = 22)	0 (0/22)	22.7 (5/22)	–
Charcuterie products (*n* = 20)	0 (0/20)	25 (5/20)	–
Raw red meat (*n* = 13)	30.7 (4/13)	38.5 (5/13)	0.007
Total (*N* = 500)	5 (25/500)	27.2 (136/500)	≤0.001

^∗^χ^2^*test and Fisher’s exact 2-tailed test analysis were performed and differences were considered significant at values of *P* ≤ 0.05*.

**Table 4:** Prevalence of different *Salmonella* serovars isolated from different types of food samples.

Type of samples	Types of isolated serovars	Number of serovars
Raw poultry meat	*Salmonella* Zanzibar	2
	*Salmonella* kentucky	2
	*Salmonella* Manchester	1
	*Salmonella* Schwarzengrund	1
	*Salmonella* Bredeney	1
	*Salmonella* Altona	1
		
Raw red meat	*Salmonella* Anatum	2
	*Salmonella* Amsterdam	1
	*Salmonella* Altona	1
		
Sea food (fish)	*Salmonella* kentucky	5
	*Salmonella* Schwarzengrund	1
		
Milk	*Salmonella* Altona	1
	*Salmonella* Anatum	3
	*Salmonella* Manchester	2
		
Raw vegetable salad	*Salmonella* Bredeney	1
		Total *N* = 25

### Detection of *Salmonella* spp. by qPCR Assay

The developed TaqMan^®^ qPCR assay including newly designed primer and probe sets for *Salmonella* spp. *invA* gene detection was used on the genomic DNA extracted from a pre-enrichment food samples medium and to confirm the identification of the 25 isolated *Salmonella* spp. QPCR results were compared to culture and biochemical results (**Table 3**). qPCR showed a specific amplification corresponding to *Salmonella* spp. for all species identified biochemically and for 136 out of 500 food samples (27.2%). *InvA* DNA was detected in 25 of 25 *Salmonella* positive specimens (100% sensitivity) and in 111 of 475 *Salmonella* culture negative food samples (23.4%) by qPCR (**Table 3**). Among the 475 culture negative food samples, 364 were negative by the qPCR (76.6% specificity). The comparison between the positivity of *Salmonella* spp. detection by conventional culture and qPCR methods showed a difference statistically significant (*P* ≤ 0.001) (**Table 3**).

As shown in **Table 3**, qPCR results yielded high rates of *Salmonella* spp. positivity ranging from 24 to 60%: i.e., in raw poultry meat 27/45 (60%) and in milk samples 31/93 (33.3%) as well as in raw red meat 5/13 (38.5%) and in fish (11/46, 23.9%). Results were statistically significant compared to *Salmonella* culture positivity. The prevalence of *Salmonella* spp. in cakes, dairy products, cooked meals, charcuterie products using qPCR was 11/41 (26.8%), 5/22 (22.7%), 32/150 (21.3%), and 5/20 (25%), respectively, compared to 0% as demonstrated by culture for all the examined samples (**Table 3**). Fresh fruit and vegetables were positive for *Salmonella* spp. in 9/70 (12.8%) vs .1/70 (1.4%) by qPCR and culture, respectively.

## Discussion

Salmonellosis is one of the most prevalent anthropozoonotic infections posing a serious health risk. Poultry or poultry products pose the greatest risk of *Salmonella* food contamination (Ashton, 1990; Coburn et al., 2007). However, *Salmonella* could also be present in other types of raw or cooked food, beverages and milk which can be pathogenic to humans (Ashton, 1990; Coburn et al., 2007). Therefore, the ability to control bacterial pathogens in foods is very relevant, in particular the early detection of *Salmonella* contamination.

Though the prevalence of *Salmonella* in food has been mentioned previously in Tunisia, all studies have focused solely on positive culture isolates (Fendri et al., 2013; Oueslati et al., 2016). Therefore, there is no study which evaluated qPCR as a screening tool to investigate the presence of *Salmonella* spp. DNA directly on pre-culture enrichment broths of 500 different commercialized food matrices.

Our results showed that qPCR was able to increase the detection level of *Salmonella* spp. in different food matrices in up to 27.2% compared to 5% obtained by conventional culture methods (*P* ≤ 0.001). Data from 25 out of the 500 tested food samples were in agreement by both methods giving a relative sensitivity of 100% for our qPCR. In addition, 111 of 475 (23%) *Salmonella* negative food samples as evaluated by the traditional cultural and biochemical methods were *invA* gene positive using the qPCR assay. The remaining 364 food samples were negative for *Salmonella* by both methods showing a relative qPCR specificity of 76.6 %. Conventional culture methods detected *Salmonella* DNA in 1.4% of our selected fresh fruit and vegetables samples and qPCR detected evidence of *Salmonella* infections in 12.8 % of them. Therefore, our study also tested the performance of *invA* qPCR detection for *Salmonella* in fresh products known with the occurrence of inhibitory substances (Delbeke et al., 2015). No inhibitory effect was detected in food samples, as demonstrated by the IAC control amplification results that allows to avoid false-negative results due to PCR inhibitors and the loss of DNA during sample processing (Malorny et al., 2004; Schrader et al., 2012; D’Agostino et al., 2015; Kasturi and Drgon, 2017).

The positivity of samples with negative culture by qPCR could be explained by the alteration of cells targets even though the food was pre-enriched overnight, i.e., they were viable but non-culturable. It was reported that to distinguish *Salmonella* from the other bacteria that are growing on the selective solid agar, the number of target cells must exceed 10^4^ CFU/ml after enrichment in tetrathionate broth (TTB) (Löfström et al., 2004; O’Regan et al., 2008). Accordingly, *Salmonella* may have survived within food in sufficient number to cause the clinical symptoms even though the bacteria was not detected by laboratory classical methods (Hein et al., 2006; González-Escalona et al., 2009; Fallschissel et al., 2009; Zheng et al., 2014; Delbeke et al., 2015). In addition, it was reported that the pre-enrichment step, dilute the number of dead cells, thus increasing the ability of qPCR to detect viable cells (Schrank et al., 2001; González-Escalona et al., 2009; Zheng et al., 2014; D’Agostino et al., 2015). However, *Salmonella* detection by qPCR was not evaluated using *invA* mRNA as target, and this is a study limitation. Thus, the amplification of DNA from dead *Salmonella* cells could also be occurred and reconcile the fact of the high *Salmonella* spp. qPCR positivity in culture negative samples. On the other hand, food contamination with *Salmonella* may be underestimated using culture technique when food contained natural compounds with antimicrobial properties that could interfere with *Salmonella* detection (Jean-Gilles Beaubrun et al., 2016). In a study by Jean-Gilles Beaubrun et al. (2016), the results showed that pre-enrichment broths supplemented with 2% (vol/vol) corn oil is an interesting alternative to improve the recovery of *Salmonella* by 50% from dried food. In fact, corn oil can neutralize the effect of the antimicrobial compounds present in food without compromising the growth of *Salmonella* (Jean-Gilles Beaubrun et al., 2016). However, similar studies should be performed for any food matrices since they differed in their organic compounds.

The qPCR assay targeting *InvA* region showed a good specificity and no cross-amplification with sequences from *E. coli, Shigella* spp. and other enterobacterial pathogens was shown. In addition, all selected *Salmonella* serotypes could be amplified. Indeed, qPCR and extraction controls consistently yielded negative results confirming that there are no false-positivent findings and the *invA* amplification is derived solely from food associated *Salmonella* spp. The qPCR assay exhibited a good intra and inter-run reproducibility. The threshold limit was around 2 copies of the *invA* gene per PCR which is similar to that of the qPCR developed previously by Hein et al. (2006) and more sensitive than those previously reported by Malorny et al. (2007) (10 copies/PCR) and by Singh and Mustapha (2013) (100 genome equivalents of *Salmonella*/PCR). Indeed, a positive result at the lowest tested contamination level of *Salmonella* spp. in food was obtained (8.5 × 10^0^ CFU/ml) by our qPCR assay. The qPCR sensitivity was comparable to that obtained with several in house qPCR targeting the *Salmonella* spp. *invA* region (González-Escalona et al., 2009; Hyeon et al., 2010; Zheng et al., 2014). The variation of the detection limit in the reported different assays could be due to the different types of food matrices used and the inhibitory factors of each type of food. Thus, a good sensitivity is required when objectives are particularly the detection of low bacterial copies and the dilution of PCR inhibitors substances possibly derived from food matrix.

Another pivotal technical aspect is the efficiency of the selected lysis-GuSCN DNA extraction method which was recommended previously by Kawasaki et al. (2010) for its good reproducibility, the capacity to remove inhibitors mainly from pre-enrichment media and its low cost compared to the commercially used methods (Uttendaele et al., 2000; Warren et al., 2007; Elizaquivel and Aznar, 2008; Kawasaki et al., 2010).

In this study, *S.* Anatum is the serovar most commonly found in raw red meat. *S*. Typhimurium, known by its clinical importance, is commonly isolated in the beef industry and is a highly prevalent serotype in outbreaks both in the US and in Asian countries (O’Regan et al., 2008; Zheng et al., 2014). However, this serotype was not detected in our range of food. Recently, a Tunisian study have showed that *S*. Typhimurium is seldom found in Tunisia but *S*. Montevideo is the dominant serovar isolated in beef products (Oueslati et al., 2016). Indeed, *S.* Kentucky was the most frequent serotype isolated from fish and raw poultry meat. *S*. Enteritidis, the most isolated serotype in chicken industry and food poisoning was also not found in our samples. Our results are also different from those previously published in which *S*. Indiana, *S.* Infantis, *S*. Agona, *S*. Senftenberg, and *S.* Enteritidis were demonstrated to be the most common *Salmonella* serotypes in chicken (Wang et al., 2013; Yang et al., 2013; Dong et al., 2014). This disparity can be attributed to the difference in the sampling sources involved in these studies as well as the seasonal variation of some serovars. Indeed, geographical and climatic differences may also contribute to these differences (Wang et al., 2013; Yang et al., 2013; Dong et al., 2014). In Tunisia, Fendri et al. (2013) showed fairly significant levels of contamination of broilers with *S*. Enteritidis and *S*. Kentucky but they indicated that London and Irena serovars are the most isolated from clam, i.e., sea food but no *S.* Kentucky contamination.

## Conclusion

Our study is the first to use a combined enrichment/*invA* qPCR method as a screening assay to detect *Salmonella* DNA in different food matrices in Southern Tunisia. QPCR results indicate that *Salmonella* contamination is common in milk and in different types of commercialized food.

## Author Contributions

MS: designed, accomplished the experiment, interpreted the results, and wrote the article manuscript. AB: participated in the study design, accomplished the statistical analysis, interpreted the results, and revised critically the manuscript. SS, SH, CM, and SK: participated in all laboratory analysis and revised critically the manuscript. RG: revised the statistical analysis of q-PCR data and revised critically the manuscript. FM-A: supervised the experimental work, interpretation of results, and revised critically the manuscript.

## Conflict of Interest Statement

The authors declare that the research was conducted in the absence of any commercial or financial relationships that could be construed as a potential conflict of interest.

## Footnotes

1 http://primer3.wi.mit.edu

2 http://www.ncbi.nlm.nih.gov/
